# Model-based optimization of peep, a strategy and its implementation

**DOI:** 10.1186/2197-425X-3-S1-A681

**Published:** 2015-10-01

**Authors:** SE Rees, DS Karbing

**Affiliations:** Health Science and Technology, Aalborg University, Aalborg, Denmark

## Introduction

The Beacon Caresystem (Mermaid Care, Denmark) is a decision support system to advise on appropriate ventilator settings, based upon mathematical physiological models, tuned to the individual patient (1). A recent development in this system is the addition of mathematical models of the effects of PEEP, enabling the system to provide advice on PEEP.

## Objectives

This abstract describes the mathematical models applied in the Beacon Caresystem to optimize PEEP level.

## Methods

Mathematical models were built to describe two aspects of the effects of PEEP 1) the modification of ventilation/perfusion (V/Q) matching and lung mechanics, and 2) the effects of PEEP on supporting respiratory muscle load. These models were integrated with those already in the system, i.e. of pulmonary gas exchange, lung mechanics, acid-base chemistry, and respiratory drive to enable simulation of the patient specific effects of PEEP.

## Results

Model implementations are illustrated below. These models represent baseline conditions, with initial values of gradients (slope_i_) adapted automatically according to patient response to changes in PEEP. Some initial gradients are relative, for example the gradient for changes in shunt (A) is a function of the shunt value, with greater reduction in shunt expected on increasing PEEP for high values of shunt. A-C illustrate V/Q and compliance changes. High V/Q is represented as a modified arterial to end tidal CO_2_ gradient. D and E represent changes in tidal volume (VT) with PEEP. The models are implemented to account for three situations: i) where too little PEEP may result in high VT; ii) where too much PEEP may result in low VT possibly due to diaphragm over-distention; and iii) where too little PEEP may result in low VT.

## Conclusions

The Beacon Caresystem includes a novel model-based strategy for setting PEEP, including both the need to optimize pulmonary function and respiratory muscle load.

## Grant Acknowledgment

SER and DSK are minor shareholders and perform consultancy work for Mermaid Care.

**Figure 1:**
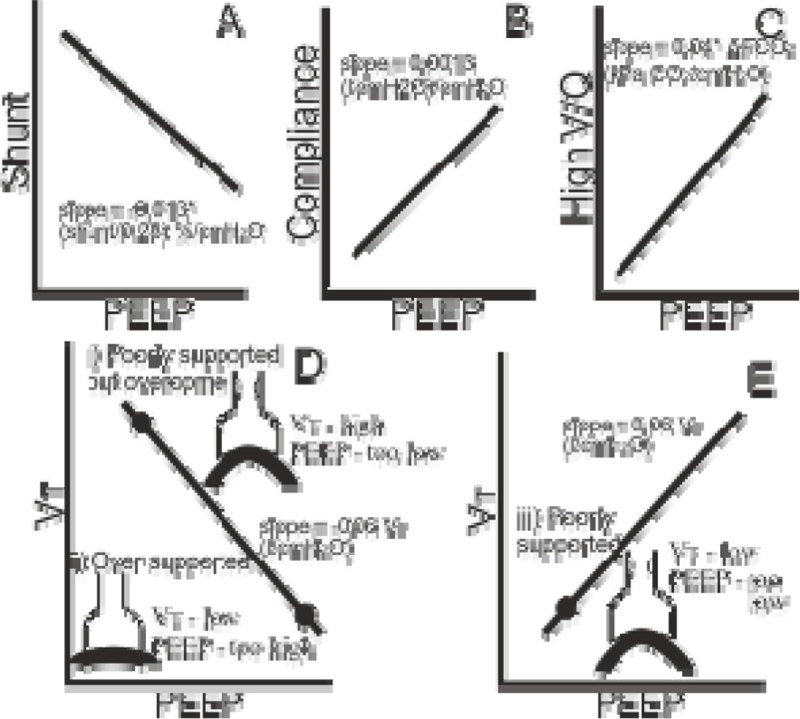
**PEEP models.**

## References

[1] Rees SE (2011). The Intelligent Ventilator (INVENT) project: The role of mathematical models in translating physiological knowledge into clinical practice. Computer Methods and Programs in Biomedicine.

